# Association between balance ability and cardiovascular disease onsets among middle-aged and older adults: an observational cohort study from the China Health and Retirement Longitudinal Study

**DOI:** 10.3389/fpubh.2025.1436520

**Published:** 2025-01-24

**Authors:** Yinghe Lin, Shanshan Zhong, Zhihua Sun

**Affiliations:** Department of Endocrinology, The Affiliated Panyu Central Hospital, Guangzhou Medical University, Guangzhou, China

**Keywords:** postural balance, public health, cardiovascular diseases, heart disease risk factors, stroke

## Abstract

**Background:**

Previous studies showed the association between poor balance ability and a high risk of cardiovascular disease (CVD) mortality. However, there is little evidence regarding balance function and the onset of CVD. We aimed to examine the relationship between the balance ability and incident CVD risk.

**Methods:**

This study retrospectively included participants (≥45 years) without CVD at baseline from the China Health and Retirement Longitudinal Study (CHARLS) in 2011 and those who were followed up in 2018. CHARLS used the semi-tandem stand test to assess balance ability. CVD was defined as the presence of physician-diagnosed heart disease and/or stroke. Cox proportional hazards models (considering competing risks) and stratification analysis were used to determine the association between balance ability and incident CVD risk.

**Results:**

The median age of the 10,154 participants was 57.0 (51.0, 64.0) years old, with 51.0% female. Compared to those with good balance ability, individuals with moderate balance ability had a higher risk of incident CVD [HR (95% CI): 1.15 (1.03–1.28)], and the risk was more pronounced in female (20.0%), rural residence (21.0%), never smoking (22.0%), never drinking (23.0%), hypertension (16.0%), without dyslipidemia (17.0%), and without kidney disease (13.0%) participants. After multiple imputations of metabolic biomarkers data, the results of sensitivity analyses were generally consistent.

**Conclusion:**

Lower balance ability was associated with an increased risk of incident CVD among middle-aged and older Chinese adults. The simple, economical, effective, and safe physical measurements of balance function deserve further investigation in public health.

## 1 Introduction

Standing balance depends on a complex set of sensorimotor control systems (1). Excellent balance ability needs correct inputs (from the vestibular, visual, and proprioceptive systems), integration (by the central nervous system), and motor output (by the musculoskeletal system). As a component of physical function evaluation (2, 3), lots of previous studies have reported that poor balance ability was associated with a high risk of falls (4, 5), which is the second leading cause of death by unintentional injury worldwide (6). Meanwhile, recent studies have shown a concrete negative relationship between standing balance ability and death from all causes (7–10). Still, the measurements of balance ability were not uniform (7), including the semi-tandem stand test (3), the modified Romberg test (9), and the one-legged stance test (8, 10), and others.

Cardiovascular disease (CVD) is the first leading cause of death worldwide, significantly impacting the well-being of individuals, families, and societies (11). Compared with traditional clinical risk factors of CVD, such as cholesterol and blood glucose, physical function assessments do not require blood assays and cost less medical resources. To improve the public health burden of CVD, researchers have found that simple, inexpensive, and safe assessments of physical function were promising as new predictors of the risk of CVD, including walking speed (12), grip strength (13), standing balance ability (8, 9), and comprehensive scales (14, 15).

For now, among different assessments of physical function, poor balance ability has been found to be associated with a high risk of CVD mortality in the older adult Japanese population (8) and the middle-aged and older American population (9). The former used the one-legged standing test, while the latter used the modified Romberg test. However, there is insufficient evidence regarding balance function and the onset of CVD.

In this context, to complement the value of the balance test in public health, it is necessary to investigate the relationship between the standing balance ability and CVD risk in various conditions. Therefore, in this study, we used the nationally representative data from the China Health and Retirement Longitudinal Study (CHARLS) (16) to conduct longitudinal analyses to investigate whether the standing balance ability developed by CHARLS can predict the risk of CVD in middle-aged and older Chinese population.

## 2 Materials and methods

### 2.1 Study population

This study used data from the CHARLS, an ongoing nationally representative survey of middle-aged and older adult individuals in China. Briefly, the CHARLS collects data through face-to-face interviews, using a structured questionnaire, from a nationally representative sample of the Chinese population aged ≥45 years, selected using multistage stratified probability-proportionate-to-size sampling. The survey mainly collects data on sociodemographics variables, lifestyle-related factors, and health-related information. Besides, the CHARLS includes various physical measurements and blood sample collection. The baseline survey was conducted in 2011, and all participants were followed up every 2–3 years.

In the longitudinal analysis, this study selected the data from the CHARLS 2011 (wave 1) to 2018 (wave 4) covering for 2013 (wave 2) and 2015 (wave 3). 17,708 participants who were enrolled in the wave 1 were excluded due to following reasons: (1) age < 45 years (*n* = 777); (2) missing information on assessment of standing balance ability (*n* = 4,514); (3) missing self-reported information on CVD in wave 1 (*n* = 26); (4) having CVD in wave 1 (*n* = 1782); (5) missing self-reported information on CVD during follow-up (*n* = 455). Finally, 10,154 individuals were eligible for this longitudinal analysis, including 9,508 (93.6%) followed up in the wave 2, 9,285 (91.4%) followed up in the wave 3, and 8,744 (86.1%) followed in the wave 4. Six hundred eighty people (6.7%) were followed up once, 1,565 people (15.4%) were followed up twice and 7,909 people (77.9%) were followed up three times.

### 2.2 Assessment of standing balance ability

All participants started with a semi-tandem standing test, a posture that needs the heel side of one foot touching the big toe of the other. Those who failed to hold the semi-tandem standing test for 10 s were classified as having poor balance ability. Those who could hold the semi-tandem standing test for 10 s continued to try the full-tandem standing test, which needed to stand with the heel of one foot touching the toes of the front of the other foot. During the full-tandem standing test, participants aged ≥70 years who lasted ≥30 s and <70 years who lasted ≥60 s were classified in the group of good balance ability. If the participants could pass the semi-tandem standing test but not the full-tandem standing test, they were classified in the group of moderate balance ability. All participants were allowed to use their arms, bend their knees, or move their bodies to maintain balance during all the balance ability tests.

### 2.3 Definition of CVD events

In accordance with the CHARLS research manual (16) and previous studies (13, 17), the definition of CVD was established through interviews with participants and recorded information using structured questionnaires in our study. Participants with CVD was defined as the presence of a diagnosis by a doctor of heart attack, coronary heart disease, angina, congestive heart failure, other heart problems, or stroke. Additionally, CVD was also identified when participants were taking medication for heart problems and their complications or stroke and its complications. The specific questionnaire questions are as follows:

1. “Have you been diagnosed with heart attack, coronary heart disease, angina, congestive heart failure, or other heart problems by a doctor?”;
2. “Have you been diagnosed with stroke by a doctor?”;
3. “Are you now taking any treatments (for example, Chinese traditional medicine, Western modern medicine, or other treatments) to treat heart problems or their complications?”;
4. “Are you now taking any treatments (for example, Chinese traditional medicine, Western modern medicine or other treatments) to treat stroke or its complications?”

### 2.4 Covariates

In the CHARLS, volunteers used a standardized and structured questionnaire to collect information on demographic factors (including age and sex), residence (urban or rural), education level (less than lower secondary, upper secondary or vocational training, or tertiary), and health behaviors (including the history of smoking and drinking) and past medical history. Past medical history included hypertension, diabetes, dyslipidemia, kidney disease, and whether using modern medications for them. Self-reported physician diagnoses defined all past medical records (18). In addition, hypertension was assessed as mean systolic blood pressure (SBP) ≥140 mm Hg and/or mean diastolic blood pressure (DBP) ≥90 mm Hg (19); diabetes was diagnosed as fasting plasma glucose (FPG) ≥7.0 mmol/L, random plasma glucose (RPG) ≥11.1 mmol/L and/or glycosylated hemoglobin (HbA1c) ≥6.5% (20); dyslipidemia was defined as total cholesterol (TC) ≥240 mg/dL, triglycerides (TG) ≥200 mg/dL, low-density lipoprotein cholesterol (LDL-C) ≥160 mg/dL and/or high-density lipoprotein cholesterol (HDL-C) <40 mg/dL (21). In our study, the body mass index (BMI, kg/m^2^) was considered and overweight was defined as a BMI ≥25 kg/m^2^. The estimated glomerular filtration rate (eGFR, mL/min/1.73 m^2^) was based on the creatinine equation (22).

### 2.5 Statistical analysis

This study described continuous variables as mean ± standard deviation (SD) or median and interquartile range (IQR), while categorical variables were presented as frequencies and percentages. First, differences in baseline characteristics of all participants between the two groups by outcome of CVD (incident CVD and non-incident CVD) were compared using independent two-sample *t*-test, chi-square test, Fisher’s exact test, or Mann–Whitney *U* test, as appropriate. Second, in the longitudinal analysis, our group calculated the incidence rates of CVD per 1,000 person-years in CHARLS 2011 to 2018 and used Cox proportional hazards models considering competing risks (non-CVD-related deaths) to assess the association between standing balance ability and incident CVD by hazard ratio (HR) with 95% confidence interval (CI). The follow-up time and the time of incident CVD were determined according to each individual’s interview time and questionnaire results in the CHARLS database. In the longitudinal analysis, as the primary variable, balance ability with four progressive models were introduced: Model 1, adjusted for age and sex; Model 2, additionally adjusted for residence, education level, and history of smoking and drinking; Model 3, additionally adjusted overweight, SBP, and DBP; and Model 4, additionally adjusted for history of comorbidities and medication use. Based on Model 4, we performed stratification analysis by age, sex, overweight, smoking, drinking, and comorbidities.

Under the assumption of random missing values, the multiple imputations selected in this study addressed missing metabolic biomarkers data, which used chained equations for interpolation. As the sensitivity analysis (Supplementary material), metabolic biomarkers such as plasma glucose, HbA1c, TC, TG, LDL-C, HDL-C, and eGFR were additionally adjusted in Model 5.

For this study, the statistical analyses were conducted using Stata 17.0 (Stata Corp.) and SPSS 23.0 (IBM Corp.). Two-sided *p*-values <0.05 were indicative of statistical significance for all analyses.

## 3 Results

### 3.1 Baseline characteristics of all participants by the outcome of CVD

The median age of the 10,154 middle-aged and older participants was 57.0 (51.0, 64.0) years old, with 51.0% female. The median age of the 7,554 excluded participants was 58.0 (49.0, 67.0) years old, with 53.6% female. A detailed comparison of included and excluded participants is shown in Supplementary Table S1.

The prevalence of individuals with incident CVD and without incident CVD during follow-up was 17.4% (1767/10154) and 82.6% (8,387/10154), respectively. A total of 8,023 (79.0%) participants passed all the balance tests and were classified as having good balance ability; 2032 (20.0%) finished parts of the tests and were classified as moderate balance ability; 99 (1.0%) failed in all the tests and were classified as poor balance ability.

Compared with participants without incident CVD, those with incident CVD were more likely to be older (59.0 vs. 57.0 years), female (55.8% vs. 50.0%), urban setting (37.3% vs. 34.2%), lower balance ability (74.3% vs. 80.0%) and poorer traditional CVD risk factors, including higher BMI (23.7 vs. 22.8 kg/m^2^), higher SBP (130.5 vs. 124.5 mm Hg), higher DBP (76.3 vs. 74.0 mm Hg), higher prevalence of hypertension (50.3% vs. 33.8%), higher prevalence of diabetes (14.6% vs. 10.2%), higher prevalence of dyslipidemia (39.7% vs. 30.8%), higher prevalence of kidney disease (6.7% vs. 5.2%). A detailed comparison of baseline variables for all participants according to the outcome of CVD is presented in Table 1.

**Table 1:** Baseline characteristics of all participants by the outcome of CVD.

Characteristics	Total participant (*n* = 10,154)	Incident CVD (*n* = 1767)	Non-incident CVD (*n* = 8,387)	*p*-value
Age (years)	57.0 (51.0, 64.0)	59.0 (53.0, 66.0)	57.0 (50.0, 63.0)	<0.001
Gender (%)				<0.001
Male	4,976 (49.0)	781 (44.2)	4,195 (50.0)	
Female	5,178 (51.0)	986 (55.8)	4,192 (50.0)	
Residence (%)				0.013
Urban	3,527 (34.7)	659 (37.3)	2,868 (34.2)	
Rural	6,627 (65.3)	1,108 (62.7)	5,519 (65.8)	
Education (%)				0.751
Less than lower secondary	9,119 (89.8)	1,579 (89.4)	7,540 (89.9)	
Upper secondary or vocational training	910 (9.0)	164 (9.3)	746 (8.9)	
Tertiary	125 (1.2)	24 (1.3)	101 (1.2)	
Ever/current smoke (%)^a^	4,100 (40.4)	680 (38.5)	3,420 (40.8)	0.073
Ever/current alcohol (%)^a^	4,099 (40.4)	678 (38.4)	3,421 (40.8)	0.060
BMI (kg/m^2^)^a^	23.0 (20.7, 25.5)	23.7 (21.3, 26.5)	22.8 (20.6, 25.3)	<0.001
Overweight (%)	2,930 (29.1)	652 (37.2)	2,278 (27.4)	<0.001
Blood pressure (mm Hg)^a^				
Systolic	125.5 (113.0, 140.0)	130.5 (117.4, 146.0)	124.5 (112.5, 139.0)	<0.001
Diastolic	74.0 (66.5, 82.5)	76.3 (68.5, 85.0)	74.0 (66.5, 82.0)	<0.001
Comorbidities (%)^a^				
Hypertension	3,713 (36.7)	886 (50.3)	2,827 (33.8)	<0.001
Diabetes	1,106 (11.0)	257 (14.6)	849 (10.2)	<0.001
Dyslipidemia	3,246 (32.4)	694 (39.7)	2,552 (30.8)	<0.001
Kidney disease	552 (5.5)	118 (6.7)	434 (5.2)	0.012
Modern medications for comorbidities (%)^a^				
Hypertension	1,358 (13.4)	413 (23.5)	945 (11.3)	<0.001
Diabetes	266 (2.6)	79 (4.5)	187 (2.3)	<0.001
Dyslipidemia	293 (2.9)	95 (5.5)	198 (2.4)	<0.001
Kidney disease	194 (1.9)	37 (2.1)	157 (1.9)	0.543
Metabolic biomarkers^b^				
Plasma glucose (mmol/L)	5.6 (5.2, 6.2)	5.7 (5.2, 6.3)	5.6 (5.2, 6.2)	0.002
HbA1c (%)	5.1 (4.9, 5.4)	5.2 (4.9, 5.5)	5.1 (4.9, 5.4)	<0.001
TC (mg/dL)	190.6 (167.4, 215.3)	194.1 (169.8, 219.9)	189.8 (166.6, 214.6)	0.001
TG (mg/dL)	103.5 (74.3, 150.5)	109.7 (80.5, 160.2)	101.8 (72.6, 148.7)	<0.001
LDL-C (mg/dL)	114.0 (93.2, 137.2)	116.4 (94.7, 140.3)	113.7 (92.8, 136.1)	0.007
HDL-C (mg/dL)	49.9 (41.0, 60.3)	48.7 (39.8, 58.7)	49.9 (41.0, 60.7)	<0.001
eGFR (mL/min/1.73 m^2^)	100.0 (85.5, 116.1)	98.1 (83.8, 113.7)	100.2 (85.8, 116.8)	0.001
Balance test (%)				<0.001
Good	8,023 (79.0)	1,313 (74.3)	6,710 (80.0)	
Moderate	2032 (20.0)	435 (24.6)	1,597 (19.0)	
Poor	99 (1.0)	19 (1.1)	80 (1.0)	
Incident CVD (%)				–
Heart disease	1,330 (13.2)	–	–	
Stroke	578 (5.7)	–	–	

Data are shown as mean ± standard deviation, median (interquartile range), or numbers (percentages).

^aMissing data: 1 for smoking, 5 for drinking, 81 for BMI, 75 for blood pressure, 32 for hypertension, 77 for diabetes, 130 for dyslipidemia, 49 for kidney disease, 52 for hypertension medications, 86 for diabetes medications, 180 for dyslipidemia medications, 51 for kidney disease medications.^

^bAmong the measurements of metabolic biomarkers, 616 participants were non-fasting; 2,741 without the measurement of plasma glucose, 2,679 without HbA1c, 2,735 for TC, 2733 for TG, 2743 for LDL-C, 2729 for HDL-C, 2744 for eGFR.^

CVD, cardiovascular disease; BMI, body mass index; HbA1c, glycosylated hemoglobin; TC, total cholesterol; TG, triglyceride; LDL-C, low-density lipoprotein cholesterol; HDL-C, high-density lipoprotein cholesterol, eGFR, the estimated glomerular filtration rate.

### 3.2 Longitudinal analysis between baseline balance test and incident CVD

During the 7.0 (6.9–7.0) years of follow-up, 1,767 participants developed the CVD outcome (1,330 heart disease and 578 stroke cases). In the longitudinal analysis, the incidence rate of CVD was 26.1 per 1,000 person-years among individuals with good balance ability, 35.2 per 1,000 person-years among those with moderate balance ability, and 34.0 per 1,000 person-years among those with poor balance ability.

The result of the baseline balance test demonstrated significant associations with CVD outcomes in all models (Table 2). After accounting for the most covariates in Model 4, individuals with moderate balance ability [HR (95% CI): 1.15 (1.03–1.28)] had higher risks of new-onset CVD than those with good balance ability. A detailed longitudinal association between baseline characteristics and incident CVD in Model 4 is shown in Supplementary Table S2. For CVD components, individuals with moderate balance ability [1.16 (1.02–1.32)] were more likely to develop heart disease than those with good balance ability in Model 4, but not to stroke. However, a similarly significant difference incidence rate between individuals with poor and good balance ability was not found (Table 2).

**Table 2:** Incidence of CVD according to baseline balance test, 2011–2018.

Outcome	No. events	Incidence rate, per 1,000 person-years	HR (95% CI)			
			Model 1^a^	Model 2^b^	Model 3^c^	Model 4^d^
CVD			(*n* = 10,154)	(*n* = 10,148)	(*n* = 9,993)	(*n* = 9,750)
Good	1,313	26.1 (24.7, 27.5)	Reference	Reference	Reference	Reference
Moderate	435	35.2 (32.0, 38.6)	1.19 (1.07, 1.32)^**^	1.21 (1.09, 1.34)^**^	1.18 (1.05, 1.31)^**^	1.15 (1.03, 1.28)^*^
Poor	19	34.0 (21.7, 53.2)	0.87 (0.55, 1.40)	0.87 (0.54, 1.39)	0.89 (0.55, 1.43)	0.87 (0.53, 1.44)
Heart disease			(*n* = 10,110)	(*n* = 10,104)	(*n* = 9,949)	(*n* = 9,732)
Good	983	19.6 (18.4, 20.9)	Reference	Reference	Reference	Reference
Moderate	333	27.0 (24.3, 30.1)	1.21 (1.07, 1.36)^**^	1.23 (1.09, 1.39)^**^	1.20 (1.06, 1.36)^**^	1.16 (1.02, 1.32)^*^
Poor	14	25.0 (14.8, 42.3)	0.86 (0.50, 1.47)	0.86 (0.50, 1.47)	0.87 (0.50, 1.50)	0.82 (0.46, 1.45)
Stroke			(*n* = 10,145)	(*n* = 10,139)	(*n* = 9,984)	(*n* = 9,744)
Good	429	8.3 (7.5, 9.1)	Reference	Reference	Reference	Reference
Moderate	143	11.1 (9.4, 13.1)	1.22 (1.02, 1.47)^*^	1.24 (1.03, 1.49)^*^	1.18 (0.98, 1.43)	1.17 (0.97, 1.42)
Poor	6	10.1 (4.5, 22.4)	0.88 (0.41, 1.90)	0.87 (0.40, 1.89)	0.90 (0.41, 1.97)	0.93 (0.42, 2.06)

^aModel 1 was adjusted for age and sex.^

^bModel 2 was adjusted for age, sex, residence, education level, smoking and drinking.^

^cModel 3 was adjusted for age, sex, residence, education level, smoking, drinking, overweight, SBP and DBP.^

^dModel 4 was adjusted for age, sex, residence, education level, smoking, drinking, overweight, SBP, DBP, comorbidities and medication use.^

*p < 0.05, **p < 0.01, ***p < 0.001.

CVD, cardiovascular disease; HR, hazard ratio; 95% CI, 95% confidence interval; SBP, systolic blood pressure; DBP, diastolic blood pressure.

In sensitivity analysis, broadly consistent results are observed after adjusting for additional metabolic biomarkers in Model 5 (Supplementary Table S3). Between groups of moderate and good balance ability, lower balance ability was associated with a significantly increased risk for composite CVD outcome [1.14 (1.02–1.27)] and the heart disease subgroup [1.15 (1.01–1.31)], but not with the stroke subgroup. Individuals with poor balance ability were not significantly associated with an higher risk of CVD outcome than those with good balance ability. Supplementary Table S4 shows other detailed longitudinal analysis between baseline characteristics and incident CVD in Model 5.

### 3.3 Stratification analysis between baseline balance test and incident CVD

**Figure 1:**
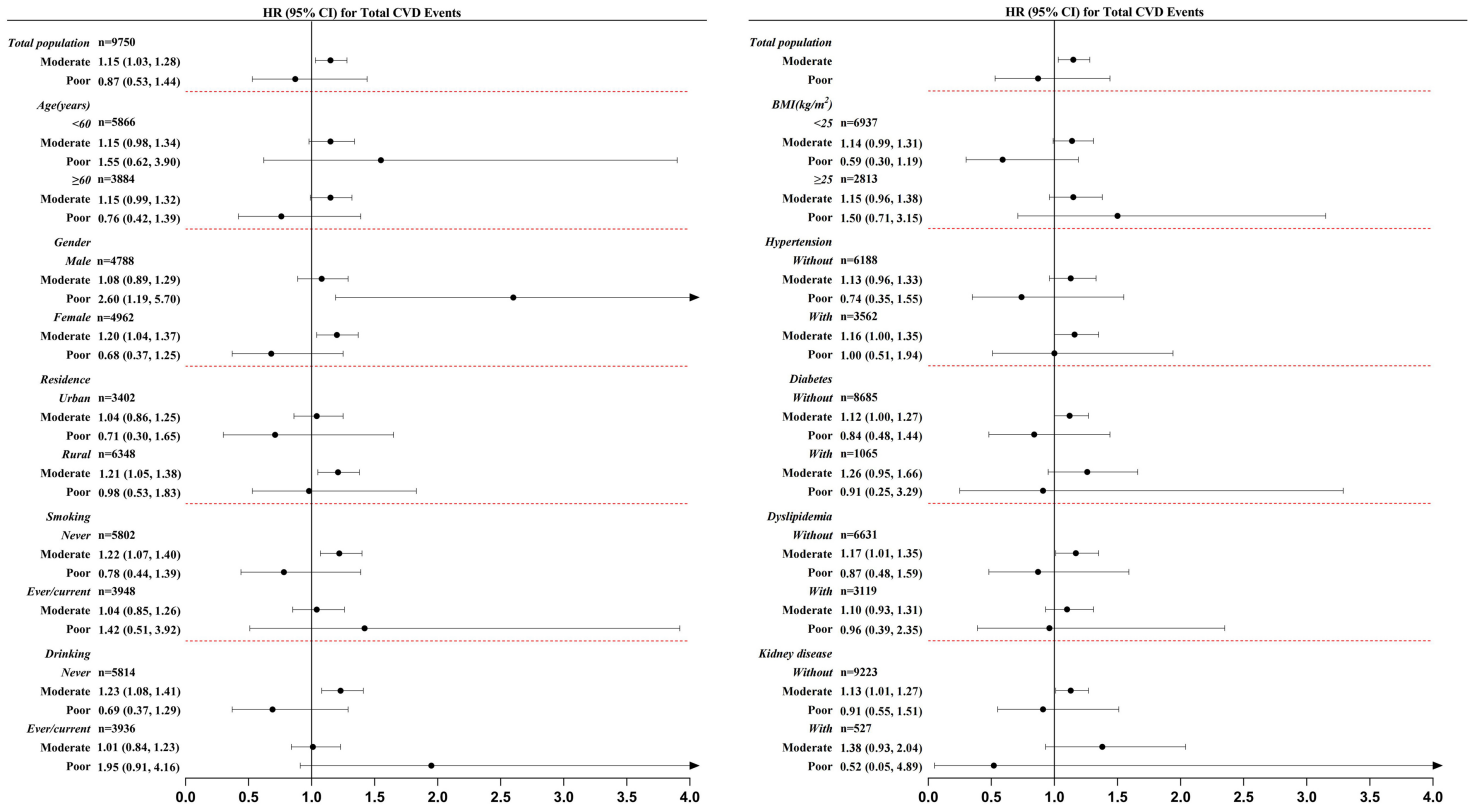
Hazard ratio (HR) and 95% CI for associations of balance ability (comparing moderate and poor level to good level) and total CVD events by baseline characteristics. The analysis was adjusted for age, sex, residence, education level, smoking, drinking, overweight SBP, DBP, comorbidities and medication use. The detailed data is presented in Supplementary Table S4. CVD, cardiovascular disease; HR, hazard ratio; 95% CI, 95% confidence interval; BMI, body mass index; SBP, systolic blood pressure; DBP, diastolic blood pressure.

In stratified analyses of Model 4 (Figure 1), individuals with moderate balance ability were associated with increased risk of CVD incidence compared to those with good balance ability among the group of female [1.20 (1.04, 1.37)], rural residence [1.21 (1.05, 1.38)], never smoking [1.22 (1.07, 1.40)], never drinking [1.23 (1.08, 1.41)], hypertension [1.16 (1.00, 1.35)], without dyslipidemia [1.17 (1.01, 1.35)], and without kidney disease [1.13 (1.01, 1.27)]. Between the subgroup of poor balance ability and good balance ability, males with lower balance ability had a 2.60 times higher risk for CVD [2.60 (1.19, 5.70)]. Other detailed stratification analysis between the baseline balance test and composite CVD and its components is presented in Supplementary Table S5.

The results were widely consistent with the above analyses after further adjusting for metabolic biomarkers in sensitivity analysis. Among the group of female [1.20 (1.04, 1.37)], rural residence [1.19 (1.04, 1.37)], never smoking [1.22 (1.07, 1.40)], never drinking [1.22 (1.07, 1.40)], without dyslipidemia [1.15 (1.00, 1.33)], and without kidney disease [1.13 (1.00, 1.26)], individuals with moderate balance ability were more strongly associated with CVD risk compared with good balance ability. Male [2.62 (1.18, 5.78)] participants with poor balance ability had increased risk of CVD than good balance ability, respectively. Other detailed stratification analysis of Model 5 is shown in Supplementary Table S6.

## 4 Discussion

In this observational cohort of a nationally representative Chinese population aged ≥45 years with 7.0 years follow-up, impaired balance ability was associated with an increased risk of CVD. These associations were slightly significant but persisted after adjusting for sociodemographic characteristics, lifestyle factors, chronic comorbidities, and metabolic biomarkers. In this study, individuals performing moderate balance ability had a 15.0% increased risk of incident CVD compared to those performing good balance ability, and the risk was more pronounced in female, rural residence, never smoking, never drinking, hypertension, without dyslipidemia, and without kidney disease participants. For CVD components, the associations between balance disorder and heart disease were stronger than stroke. Considering previous studies (8, 9, 23), these findings may help further determine the correlation between balance ability and CVD events in different populations, and those simple, economical, effective, and safe physical measurements deserve further investigation, which is conducive to public health.

Balance ability, an essential physical measurement, is associated with death from all causes (7–10), including those related to CVD-related mortality risk (8, 9). An earlier study found an association between the shorter one-legged standing time and higher cardiovascular mortality in a 1,085 Japanese adult cohort aged 65–89, with a follow-up of 10.5 years (8), and the participants with balance disorder had a 91% higher CVD mortality. For a median of 12.6 years follow-up, a later American study including 5,816 adults found a similar result that inadequate balance ability, measured by the modified Romberg test, was associated with a 65% increased risk of death from CVD (9). To expand the scant research on the correlation between the assessment of balance and the occurrence of CVD events, recently, Kim et al. (23) found that participants receiving the one-legged stance test with impaired balance ability had a 23% higher risk of incident CVD after adjusting for potential CVD risk factors by analyzing a Korean cohort; and the relationship for stroke was more substantial than heart disease.

To our knowledge, this study is the first to investigate the association between balance ability and future overall CVD events in a nationally representative cohort of Chinese adults, with results generally consistent with the previous study (23). Although there appears to be a slight increase in the identified risk of incident CVD (15.0%), the results were based on correction for multiple CVD risk factors, suggesting that balance ability may promisingly predict future CVD onsets in addition to being associated with the risk of CVD-related death (8, 9). It is worth noting that, unlike the Korean study (23), the association with heart disease was stronger than stroke in this study, and several likely reasons exist, including different study populations, designs, and balance assessments.

Furthermore, our group further analyzed the association of balance ability with the occurrence of CVD in individuals with different characteristics. In the primary outcome, the risk was highlighted in the female (20.0%), rural residence (21.0%), never smoking (22.0%), never drinking (23.0%), hypertension (16.0%), without dyslipidemia (17.0%), and without kidney disease (13.0%) group, which means that the balance function in these individuals may deserve greater attention in clinical care.

The association between balance and CVD could be explained from a behavioral perspective. Impaired balance can lead to future unexpected fall events (4, 5) and also to a fear of falling (24, 25), probably resulting in insufficient activity and extension of sedentary time, which are the often mentioned risk factors associated with increased risk of CVD (26, 27). Limited physical activity and increased sitting time play a physiological role in obesity, diabetes, hypertension, and dyslipidemia, which further aggravate the risk burden of CVD (28, 29).

This study has several strengths. First, our sample consisted of a nationally representative cohort of middle-aged and older adults in China, and the results were obtained through reliable statistical analysis. Second, this study is the first to investigate the relationship between balance ability and future CVD events in a nationally representative cohort of Chinese adults. The findings suggest that balance ability has the potential to predict future CVD onset. Third, by analyzing individuals with different characteristics, the study provides new insights into the association between balance ability and CVD onset. The association was found to be more pronounced in female, rural residence, never smoking, never drinking, hypertension, without dyslipidemia, and without kidney disease participants, respectively.

In addition, there are some limitations in this study. First, due to its observational nature, this study does not determine any causal inferences and investigate potential mechanisms. Second, due to the established study design of CHARLS, this study only involved Chinese adults aged ≥45 years from CHARLS, which may result in selection bias. Third, due to the published data from the CHARLS, this study used the semi-tandem stand test to assess balance ability, which has limited analogies with the non-uniform balance assessment literature (7). More prospective and intervention studies would be warranted to develop an applicable and uniform balance assessment and elucidate whether the findings of this study could help screen people at risk of CVD and suggest prevention to risk reduction of CVD.

## 5 Conclusion

The association of impaired balance ability with an increased risk of incident CVD was found in this nationally representative cohort of Chinese adults aged 45 and older. Future prospective and intervention studies are required to identify their causal relationship and confirm these findings in public health.

## Data Availability

The datasets presented in this study can be found in online repositories. The names of the repository/repositories and accession number(s) can be found at: http://charls.pku.edu.cn/.
