# Supplementary material for: Association between balance ability and cardiovascular disease onsets among middle-aged and older adults: an observational cohort study from the China Health and Retirement Longitudinal Study
Source: Front Public Health. 2025 Jan 24;13:1436520. doi: 10.3389/fpubh.2025.1436520 (PMC11802414; doi:10.3389/fpubh.2025.1436520)
Supplement: Supplementary file 1 [file Table_1.DOCX]

Supplementary Material

# Supplementary Tables

| Table S1. Baseline characteristics of the included and excluded participants | | | | | | | | |
| --- | --- | --- | --- | --- | --- | --- | --- | --- |
| Characteristics |  | The participants in wave 1  (n=17708) |  | The included participants (n=10154) |  | The excluded participants (n=7554) |  | P-value |
| Age (years)^a^ |  | 50.0 (57.0,65.0) |  | 57.0 (51.0,64.0) |  | 58.0 (49.0,67.0) |  | 0.009 |
| Gender (%)^a^ |  |  |  |  |  |  |  | <0.001 |
| Male |  | 8478 (47.9) |  | 4976 (49.0) |  | 3502 (46.4) |  |  |
| Female |  | 9228 (52.1) |  | 5178 (51.0) |  | 4050 (53.6) |  |  |
| Residence (%) |  |  |  |  |  |  |  | <0.001 |
| Urban |  | 7171 (40.5) |  | 3527 (34.7) |  | 3644 (48.2) |  |  |
| Rural |  | 10537 (59.5) |  | 6627 (65.3) |  | 3910 (51.8) |  |  |
| Education (%)^a^ |  |  |  |  |  |  |  | <0.001 |
| Less than lower secondary |  | 15545 (87.9) |  | 9119 (89.8) |  | 6426 (85.3) |  |  |
| Upper secondary or vocational training |  | 1793 (10.1) |  | 910 (9.0) |  | 883 (11.7) |  |  |
| Tertiary |  | 354 (2.0) |  | 125 (1.2) |  | 229 (3.0) |  |  |
| Ever/current smoke (%)^a^ |  | 6931 (39.5) |  | 4100 (40.4) |  | 2831 (38.2) |  | 0.004 |
| Ever/current alcohol (%)^a^ |  | 6823 (38.9) |  | 4099 (40.4) |  | 2724 (36.9) |  | <0.001 |
| BMI(kg/m^2^)^a^ |  | 23.1 (20.8,25.7) |  | 23.0 (20.7,25.5) |  | 23.5 (21.0,26.4) |  | <0.001 |
| Overweight (%) |  | 4207 (30.9) |  | 2930 (29.1) |  | 1277 (35.9) |  | <0.001 |
| Blood pressure (mm Hg)^a^ |  |  |  |  |  |  |  |  |
| Systolic |  | 126.5 (114.0,142.0) |  | 125.5 (113.0,140.0) |  | 129.0 (116.0,147.0) |  | <0.001 |
| Diastolic |  | 74.5 (67.0,83.0) |  | 74.0 (66.5,82.5) |  | 76.0 (67.5,85.0) |  | <0.001 |
| Comorbidities (%)^a^ |  |  |  |  |  |  |  |  |
| Hypertension |  | 6744 (38.5) |  | 3713 (36.7) |  | 3031 (41.1) |  | <0.001 |
| Diabetes |  | 2120 (12.2) |  | 1106 (11.0) |  | 1013 (13.8) |  | <0.001 |
| Dyslipidemia |  | 5697 (32.9) |  | 3246 (32.4) |  | 2451 (33.6) |  | 0.100 |
| Kidney disease |  | 1126 (6.5) |  | 552 (5.5) |  | 574 (7.8) |  | <0.001 |
| Modern medications for Comorbidities (%)^a^ |  |  |  |  |  |  |  |  |
| Hypertension |  | 3201 (18.3) |  | 1358 (13.4) |  | 1843 (25.0) |  | <0.001 |
| Diabetes |  | 648 (3.7) |  | 266 (2.6) |  | 382 (5.2) |  | <0.001 |
| Dyslipidemia |  | 780 (4.5) |  | 293 (2.9) |  | 487 (6.7) |  | <0.001 |
| Kidney disease |  | 415 (2.4) |  | 194 (1.9) |  | 221 (3.0) |  | <0.001 |
| Metabolic biomarkers^b^ |  |  |  |  |  |  |  |  |
| Plasma glucose (mmol/L) |  | 5.6 (5.2,6.3) |  | 5.6 (5.2,6.2) |  | 5.7 (5.2,6.3) |  | 0.002 |
| Glycosylated hemoglobin (%) |  | 5.1 (4.9,5.4) |  | 5.1 (4.9,5.4) |  | 5.1 (4.9,5.5) |  | 0.094 |
| Total cholesterol (mg/dL) |  | 189.8 (166.6,215.3) |  | 190.6 (167.4,215.3) |  | 188.3 (165.1,214.9) |  | 0.014 |
| Triglycerides (mg/dL) |  | 106.2 (75.2,156.6) |  | 103.5 (74.3,150.5) |  | 113.7 (91.6,136.5) |  | <0.001 |
| LDL-cholesterol (mg/dL) |  | 114.0 (92.8,136.9) |  | 114.0 (93.2,137.2) |  | 113.7 (91.6,136.5) |  | 0.104 |
| HDL-cholesterol (mg/dL) |  | 49.1 (39.8,59.5) |  | 49.9 (41.0,60.3) |  | 47.6 (38.7,58.4) |  | <0.001 |
| eGFR (mL/min/1.73 m^2^) |  | 99.5 (84.3,116.2) |  | 100.0 (85.5,116.1) |  | 99.0 (82.4,116.2) |  | 0.001 |
| Balance test (%)^c^ |  |  |  |  |  |  |  | <0.001 |
| Good |  | 10021 (77.5) |  | 8023 (79.0) |  | 1998 (72.2) |  |  |
| Moderate |  | 2739 (21.2) |  | 2032 (20.0) |  | 707 (25.5) |  |  |
| Poor |  | 163 (1.3) |  | 99 (1.0) |  | 64 (2.3) |  |  |

Data are shown as mean ± standard deviation, median (interquartile range), or numbers (percentages).

Differences in baseline characteristics of the included and excluded participants were compared using independent two-sample t test, chi-square test, Fisher’s exact test, or Mann-Whitney U test, as appropriate.

1. Missing data: 175 for age, 2 for gender, 16 for education, 149 for smoking, 167 for drinking, 4077 for BMI, 3968 for blood pressure, 209 for hypertension, 279 for diabetes, 383 for dyslipidemia, 259 for kidney disease, 248 for hypertension medications, 304 for diabetes medications, 507 for dyslipidemia medications, 261 for kidney disease medications.
2. Among the measurements of metabolic biomarkers, 963 participants were non-fasting; 6072 without the measurement of plasma glucose, 6002 without HbA1c, 6053 for TC, 6052 for TG, 6066 for LDL-C, 6045 for HDL-C, 6074 for eGFR.
3. 4785 participants in wave 1 did not perform the balance test.

Abbreviations: CVD, cardiovascular disease; BMI, body mass index; HbA1c, glycosylated hemoglobin; TC, total cholesterol; TG, triglyceride; LDL-C, low-density lipoprotein cholesterol; HDL-C, high-density lipoprotein cholesterol, eGFR, the estimated glomerular filtration rate.

| Table S2. Longitudinal association between baseline characteristics and incident CVD, 2011-2018^a^ | | | | | | | | | | |
| --- | --- | --- | --- | --- | --- | --- | --- | --- | --- | --- |
| Characteristics |  | Total participant  (n=10154) |  | Incident CVD  (n=1767) |  | Non-incident CVD  (n=8387) |  | HR (95% CI) |  | P-value |
| Age (years) |  | 57.0 (51.0, 64.0) |  | 59.0 (53.0, 66.0) |  | 57.0 (50.0, 63.0) |  | 1.02 (1.02, 1.03) |  | <0.001 |
| Gender (%) |  |  |  |  |  |  |  |  |  |  |
| Male |  | 4976 (49.0) |  | 781 (44.2) |  | 4195 (50.0) |  | Reference |  |  |
| Female |  | 5178 (51.0) |  | 986 (55.8) |  | 4192 (50.0) |  | 1.28 (1.11, 1.47) |  | 0.001 |
| Residence (%) |  |  |  |  |  |  |  |  |  |  |
| Urban |  | 3527 (34.7) |  | 659 (37.3) |  | 2868 (34.2) |  | Reference |  |  |
| Rural |  | 6627 (65.3) |  | 1108 (62.7) |  | 5519 (65.8) |  | 0.88 (0.80, 0.97) |  | 0.010 |
| Education (%) |  |  |  |  |  |  |  |  |  |  |
| Less than lower secondary |  | 9119 (89.8) |  | 1579 (89.4) |  | 7540 (89.9) |  | Reference |  |  |
| Upper secondary or vocational training |  | 910 (9.0) |  | 164 (9.3) |  | 746 (8.9) |  | 1.14 (0.97, 1.35) |  | 0.113 |
| Tertiary |  | 125 (1.2) |  | 24 (1.3) |  | 101 (1.2) |  | 1.21 (0.81, 1.81) |  | 0.351 |
| Ever/current smoke (%) |  | 4100 (40.4) |  | 680 (38.5) |  | 3420 (40.8) |  | 1.19 (1.04, 1.36) |  | 0.013 |
| Ever/current alcohol (%) |  | 4099 (40.4) |  | 678 (38.4) |  | 3421 (40.8) |  | 0.95 (0.84, 1.06) |  | 0.342 |
| BMI (kg/m^2^) |  | 23.0 (20.7, 25.5) |  | 23.7 (21.3, 26.5) |  | 22.8 (20.6,25.3) |  | - |  |  |
| Overweight (%) |  | 2930 (29.1) |  | 652 (37.2) |  | 2278 (27.4) |  | 1.20 (1.08, 1.33) |  | 0.001 |
| Blood pressure (mm Hg) |  |  |  |  |  |  |  |  |  |  |
| Systolic |  | 125.5 (113.0, 140.0) |  | 130.5 (117.4, 146.0) |  | 124.5 (112.5, 139.0) |  | 1.00 (1.00,1.01) |  | 0.454 |
| Diastolic |  | 74.0 (66.5, 82.5) |  | 76.3 (68.5, 85.0) |  | 74.0 (66.5, 82.0) |  | 1.01 (1.00,1.01) |  | 0.030 |
| Comorbidities (%) |  |  |  |  |  |  |  |  |  |  |
| Hypertension |  | 3713 (36.7) |  | 886 (50.3) |  | 2827 (33.8) |  | 1.21 (1.05, 1.41) |  | 0.010 |
| Diabetes |  | 1106 (11.0) |  | 257 (14.6) |  | 849 (10.2) |  | 1.05 (0.90, 1.23) |  | 0.505 |
| Dyslipidemia |  | 3246 (32.4) |  | 694 (39.7) |  | 2552 (30.8) |  | 1.18 (1.06, 1.30) |  | 0.002 |
| Kidney disease |  | 552 (5.5) |  | 118 (6.7) |  | 434 (5.2) |  | 1.47 (1.18, 1.83) |  | 0.001 |
| Modern medications for comorbidities (%) |  |  |  |  |  |  |  |  |  |  |
| Hypertension |  | 1358 (13.4) |  | 413 (23.5) |  | 945 (11.3) |  | 1.35 (1.18, 1.54) |  | <0.001 |
| Diabetes |  | 266 (2.6) |  | 79 (4.5) |  | 187 (2.3) |  | 1.28 (0.99, 1.65) |  | 0.052 |
| Dyslipidemia |  | 293 (2.9) |  | 95 (5.5) |  | 198 (2.4) |  | 1.24 (1.00, 1.53) |  | 0.045 |
| Kidney disease |  | 194 (1.9) |  | 37 (2.1) |  | 157 (1.9) |  | 0.82 (0.56, 1.20) |  | 0.306 |
| Balance test (%) |  |  |  |  |  |  |  |  |  |  |
| Good |  | 8023 (79.0) |  | 1313 (74.3) |  | 6710 (80.0) |  | Reference |  |  |
| Moderate |  | 2032 (20.0) |  | 435 (24.6) |  | 1597 (19.0) |  | 1.15 (1.03, 1.28) |  | 0.013 |
| Poor |  | 99 (1.0) |  | 19 (1.1) |  | 80 (1.0) |  | 0.87 (0.53, 1.44) |  | 0.588 |

1. Model 4 was adjusted for age, sex, residence, education level, smoking, drinking, overweight, SBP, DBP, comorbidities and medication use.

Abbreviations: CVD, cardiovascular disease; HR, hazard ratio; 95% CI, 95% confidence interval; BMI, body mass index; SBP, systolic blood pressure; DBP, diastolic blood pressure.

| Table S3. Incidence of CVD according to baseline balance test in Model 5, 2011-2018 | | | | | | |
| --- | --- | --- | --- | --- | --- | --- |
| Outcome |  | No. events |  | Incidence Rate,  per 1000 Person-Years |  | HR (95% CI) |
|  |  |  |  |  |  | Model 5^a^ |
| CVD |  |  |  |  |  | (n=9750) |
| Good |  | 1313 |  | 26.1 (24.7, 27.5) |  | Reference |
| Moderate |  | 435 |  | 35.2 (32.0, 38.6) |  | 1.14 (1.02, 1.27)^*^ |
| Poor |  | 19 |  | 34.0 (21.7, 53.2) |  | 0.95 (0.58, 1.57) |
| Heart disease |  |  |  |  |  | (n=9750) |
| Good |  | 983 |  | 19.6 (18.4, 20.9) |  | Reference |
| Moderate |  | 333 |  | 27.0 (24.3, 30.1) |  | 1.15 (1.01, 1.31)^*^ |
| Poor |  | 14 |  | 25.0 (14.8, 42.3) |  | 0.89 (0.50, 1.57) |
| Stroke |  |  |  |  |  | (n=9750) |
| Good |  | 429 |  | 8.3 (7.5, 9.1) |  | Reference |
| Moderate |  | 143 |  | 11.1 (9.4, 13.1) |  | 1.15 (0.95, 1.40) |
| Poor |  | 6 |  | 10.1 (4.5, 22.4) |  | 1.05 (0.48, 2.32) |

1. Model 5 was adjusted for age, sex, residence, education level, smoking, drinking, overweight, SBP, DBP, comorbidities, medication use and metabolic biomarkers.

* *P*<0.05; ** *P*<0.01; *** *P*<0.001.

Abbreviations: CVD, cardiovascular disease; HR, hazard ratio; 95% CI, 95% confidence interval; SBP, systolic blood pressure; DBP, diastolic blood pressure.

| Table S4. Longitudinal association between baseline characteristics and incident CVD in Model 5, 2011-2018^a^ | | | | | | | | | | |
| --- | --- | --- | --- | --- | --- | --- | --- | --- | --- | --- |
| Characteristics |  | Total participant (n=10154) |  | Incident CVD  (n=1767) |  | Non-incident CVD  (n=8387) |  | HR (95% CI) |  | P-value |
| Age (years) |  | 57.0 (51.0, 64.0) |  | 59.0 (53.0, 66.0) |  | 57.0 (50.0, 63.0) |  | 1.03 (1.02, 1.03) |  | <0.001 |
| Gender (%) |  |  |  |  |  |  |  |  |  |  |
| Male |  | 4976 (49.0) |  | 781 (44.2) |  | 4195 (50.0) |  | Reference |  |  |
| Female |  | 5178 (51.0) |  | 986 (55.8) |  | 4192 (50.0) |  | 1.29 (1.12, 1.49) |  | <0.001 |
| Residence (%) |  |  |  |  |  |  |  |  |  |  |
| Urban |  | 3527 (34.7) |  | 659 (37.3) |  | 2868 (34.2) |  | Reference |  |  |
| Rural |  | 6627 (65.3) |  | 1108 (62.7) |  | 5519 (65.8) |  | 0.87 (0.79, 0.96) |  | 0.007 |
| Education (%) |  |  |  |  |  |  |  |  |  |  |
| Less than lower secondary |  | 9119 (89.8) |  | 1579 (89.4) |  | 7540 (89.9) |  | Reference |  |  |
| Upper secondary or vocational training |  | 910 (9.0) |  | 164 (9.3) |  | 746 (8.9) |  | 1.13 (0.96, 1.34) |  | 0.152 |
| Tertiary |  | 125 (1.2) |  | 24 (1.3) |  | 101 (1.2) |  | 1.21 (0.81, 1.82) |  | 0.346 |
| Ever/current smoke (%) |  | 4100 (40.4) |  | 680 (38.5) |  | 3420 (40.8) |  | 1.21 (1.05, 1.38) |  | 0.007 |
| Ever/current alcohol (%) |  | 4099 (40.4) |  | 678 (38.4) |  | 3421 (40.8) |  | 0.95 (0.85, 1.07) |  | 0.398 |
| BMI (kg/m^2^) |  | 23.0 (20.7, 25.5) |  | 23.7 (21.3, 26.5) |  | 22.8 (20.6, 25.3) |  | - |  |  |
| Overweight (%) |  | 2930 (29.1) |  | 652 (37.2) |  | 2278 (27.4) |  | 1.20 (1.08, 1.34) |  | 0.001 |
| Blood pressure (mm Hg) |  |  |  |  |  |  |  |  |  |  |
| Systolic |  | 125.5 (113.0, 140.0) |  | 130.5 (117.4, 146.0) |  | 124.5 (112.5, 139.0) |  | 1.00 (1.00, 1.01) |  | 0.403 |
| Diastolic |  | 74.0 (66.5, 82.5) |  | 76.3 (68.5, 85.0) |  | 74.0 (66.5, 82.0) |  | 1.01 (1.00, 1.01) |  | 0.029 |
| Comorbidities (%) |  |  |  |  |  |  |  |  |  |  |
| Hypertension |  | 3713 (36.7) |  | 886 (50.3) |  | 2827 (33.8) |  | 1.21 (1.04, 1.40) |  | 0.011 |
| Diabetes |  | 1106 (11.0) |  | 257 (14.6) |  | 849 (10.2) |  | 1.04 (0.87, 1.24) |  | 0.654 |
| Dyslipidemia |  | 3246 (32.4) |  | 694 (39.7) |  | 2552 (30.8) |  | 1.18 (1.04, 1.33) |  | 0.008 |
| Kidney disease |  | 552 (5.5) |  | 118 (6.7) |  | 434 (5.2) |  | 1.49 (1.20, 1.86) |  | <0.001 |
| Modern medications for comorbidities (%) |  |  |  |  |  |  |  |  |  |  |
| Hypertension |  | 1358 (13.4) |  | 413 (23.5) |  | 945 (11.3) |  | 1.35 (1.18, 1.54) |  | <0.001 |
| Diabetes |  | 266 (2.6) |  | 79 (4.5) |  | 187 (2.3) |  | 1.24 (0.95, 1.61) |  | 0.115 |
| Dyslipidemia |  | 293 (2.9) |  | 95 (5.5) |  | 198 (2.4) |  | 1.21 (0.98, 1.50) |  | 0.083 |
| Kidney disease |  | 194 (1.9) |  | 37 (2.1) |  | 157 (1.9) |  | 0.82 (0.56, 1.21) |  | 0.314 |
| Metabolic biomarkers |  |  |  |  |  |  |  |  |  |  |
| Plasma glucose (mmol/L) |  | 5.6 (5.1, 6.3) |  | 5.7 (5.1, 6.4) |  | 5.6 (5.1, 6.3) |  | 1.00 (0.96, 1.03) |  | 0.838 |
| HbA1c (%) |  | 5.1 (4.8, 5.5) |  | 5.2 (4.9, 5.5) |  | 5.1 (4.8, 5.4) |  | 1.04 (0.97, 1.13) |  | 0.265 |
| TC (mg/dL) |  | 190.2 (167.4, 214.3) |  | 193.3 (170.5, 218.0) |  | 189.4 (167.0, 213.4) |  | 1.00 (0.99, 1.00) |  | 0.523 |
| TG (mg/dL) |  | 102.7 (68.1, 154.9) |  | 108.9 (75.4, 164.6) |  | 100.9 (67.3, 152.2) |  | 1.00 (1.00, 1.00) |  | 0.653 |
| LDL-C (mg/dL) |  | 114.0 (93.2, 137.0) |  | 116.8 (94.7, 139.9) |  | 113.7 (92.8, 136.1) |  | 1.00 (1.00, 1.01) |  | 0.395 |
| HDL-C (mg/dL) |  | 51.8 (42.2, 62.2) |  | 50.7 (41.0, 60.7) |  | 52.2 (42.5, 62.6) |  | 1.00 (1.00, 1.01) |  | 0.440 |
| eGFR (mL/min/1.73 m^2^) |  | 100.7 (85.3, 117.8) |  | 99.1 (83.7, 115.7) |  | 100.9 (85.6, 118.0) |  | 1.00 (1.00, 1.00) |  | 0.875 |
| Balance test (%) |  |  |  |  |  |  |  |  |  |  |
| Good |  | 8023 (79.0) |  | 1313 (74.3) |  | 6710 (80.0) |  | Reference |  |  |
| Moderate |  | 2032 (20.0) |  | 435 (24.6) |  | 1597 (19.0) |  | 1.14 (1.02, 1.27) |  | 0.017 |
| Poor |  | 99 (1.0) |  | 19 (1.1) |  | 80 (1.0) |  | 0.95 (0.58, 1.57) |  | 0.840 |

1. Model 5 was adjusted for age, sex, residence, education level, smoking, drinking, overweight, SBP, DBP, comorbidities, medication use and metabolic biomarkers.

Abbreviations: CVD, cardiovascular disease; HR, hazard ratio; 95% CI, 95% confidence interval; BMI, body mass index; SBP, systolic blood pressure; DBP, diastolic blood pressure; HbA1c, glycosylated hemoglobin; TC, total cholesterol; TG, triglyceride; LDL-C, low-density lipoprotein cholesterol; HDL-C, high-density lipoprotein cholesterol, eGFR, the estimated glomerular filtration rate.

| Table S5. Model 4: Stratification analysis between baseline balance test and incident CVD, 2011-2018^a^ | | | | | | | | | |
| --- | --- | --- | --- | --- | --- | --- | --- | --- | --- |
| Stratification analysis |  | CVD | |  | Heart disease | |  | Stroke | |
|  |  | HR (95% CI) | P-value |  | HR (95% CI) | P-value |  | HR (95% CI) | P-value |
| **Total population** |  | (n=9750) |  |  | (n=9732) |  |  | (n=9744) |  |
| Good |  | Reference |  |  | Reference |  |  | Reference |  |
| Moderate |  | 1.15(1.03, 1.28)^*^ | 0.013 |  | 1.16(1.02, 1.32)^*^ | 0.021 |  | 1.17(0.97, 1.42) | 0.104 |
| Poor |  | 0.87(0.53, 1.44) | 0.588 |  | 0.82(0.46, 1.45) | 0.489 |  | 0.93(0.42, 2.06) | 0.849 |
| **Age** |  |  |  |  |  |  |  |  |  |
| **<60 years** |  | (n=5866) |  |  | (n=5858) |  |  | (n=5861) |  |
| Good |  | Reference |  |  | Reference |  |  | Reference |  |
| Moderate |  | 1.15(0.98, 1.34) | 0.093 |  | 1.16(0.96, 1.40) | 0.132 |  | 1.04(0.75, 1.43) | 0.817 |
| Poor |  | 1.55(0.62, 3.90) | 0.347 |  | 1.41(0.50, 3.98) | 0.513 |  | 1.97(0.54, 7.16) | 0.304 |
| **≥60 years** |  | (n=3884) |  |  | (n=3874) |  |  | (n=3883) |  |
| Good |  | Reference |  |  | Reference |  |  | Reference |  |
| Moderate |  | 1.15(0.99, 1.32) | 0.067 |  | 1.16(0.98, 1.38) | 0.080 |  | 1.29(1.02, 1.64)^*^ | 0.032 |
| Poor |  | 0.76(0.42, 1.39) | 0.378 |  | 0.75(0.38, 1.49) | 0.414 |  | 0.71(0.25, 2.03) | 0.527 |
| **Gender** |  |  |  |  |  |  |  |  |  |
| **Male** |  | (n=4788) |  |  | (n=4780) |  |  | (n=4784) |  |
| Good |  | Reference |  |  | Reference |  |  | Reference |  |
| Moderate |  | 1.08(0.89, 1.29) | 0.440 |  | 0.97(0.77, 1.23) | 0.806 |  | 1.28(0.97, 1.70) | 0.084 |
| Poor |  | 2.60(1.19, 5.70)^*^ | 0.017 |  | 1.55(0.51, 4.69) | 0.437 |  | 4.92(1.67, 14.50)^**^ | 0.004 |
| **Female** |  | (n=4962) |  |  | (n=4952) |  |  | (n=4960) |  |
| Good |  | Reference |  |  | Reference |  |  | Reference |  |
| Moderate |  | 1.20(1.04, 1.37)^*^ | 0.010 |  | 1.27(1.09, 1.48)^**^ | 0.002 |  | 1.09(0.85, 1.42) | 0.494 |
| Poor |  | 0.68(0.37, 1.25) | 0.215 |  | 0.77(0.40, 1.48) | 0.434 |  | 0.40(0.12, 1.32) | 0.132 |
| **Residence** |  |  |  |  |  |  |  |  |  |
| **Urban** |  | (n=3402) |  |  | (n=3395) |  |  | (n=3399) |  |
| Good |  | Reference |  |  | Reference |  |  | Reference |  |
| Moderate |  | 1.04(0.86, 1.25) | 0.705 |  | 1.01(0.80, 1.26) | 0.961 |  | 1.32(0.97, 1.79) | 0.077 |
| Poor |  | 0.71(0.30, 1.65) | 0.421 |  | 0.66(0.25, 1.74) | 0.398 |  | 0.77(0.21, 2.80) | 0.697 |
| **Rural** |  | (n=6348) |  |  | (n=6337) |  |  | (n=6345) |  |
| Good |  | Reference |  |  | Reference |  |  | Reference |  |
| Moderate |  | 1.21(1.05, 1.38)^**^ | 0.007 |  | 1.25(1.07, 1.46)^**^ | 0.005 |  | 1.08(0.84, 1.38) | 0.549 |
| Poor |  | 0.98(0.53, 1.83) | 0.974 |  | 0.92(0.45, 1.89) | 0.814 |  | 0.99(0.36, 2.73) | 0.982 |
| **Smoking** |  |  |  |  |  |  |  |  |  |
| **Never** |  | (n=5802) |  |  | (n=5790) |  |  | (n=5799) |  |
| Good |  | Reference |  |  | Reference |  |  | Reference |  |
| Moderate |  | 1.22(1.07, 1.40)^**^ | 0.003 |  | 1.23(1.06, 1.44)^**^ | 0.008 |  | 1.30(1.02, 1.66)^*^ | 0.037 |
| Poor |  | 0.78(0.44, 1.39) | 0.401 |  | 0.78(0.41, 1.49) | 0.454 |  | 0.66(0.23, 1.89) | 0.440 |
| **Ever/current** |  | (n=3948) |  |  | (n=3942) |  |  | (n=3945) |  |
| Good |  | Reference |  |  | Reference |  |  | Reference |  |
| Moderate |  | 1.04(0.85, 1.26) | 0.714 |  | 1.04(0.83, 1.31) | 0.722 |  | 1.02(0.74, 1.40) | 0.914 |
| Poor |  | 1.42(0.51, 3.92) | 0.503 |  | 1.11(0.30, 4.03) | 0.880 |  | 2.22(0.61, 8.08) | 0.226 |
| **Drinking** |  |  |  |  |  |  |  |  |  |
| **Never** |  | (n=5814) |  |  | (n=5802) |  |  | (n=5811) |  |
| Good |  | Reference |  |  | Reference |  |  | Reference |  |
| Moderate |  | 1.23(1.08, 1.41)^**^ | 0.002 |  | 1.22(1.05, 1.42)^*^ | 0.010 |  | 1.29(1.01, 1.65)^*^ | 0.040 |
| Poor |  | 0.69(0.37, 1.29) | 0.240 |  | 0.82(0.43, 1.56) | 0.540 |  | 0.32(0.08, 1.34) | 0.118 |
| **Ever/current** |  | (n=3936) |  |  | (n=3930) |  |  | (n=3933) |  |
| Good |  | Reference |  |  | Reference |  |  | Reference |  |
| Moderate |  | 1.01(0.84, 1.23) | 0.883 |  | 1.06(0.84, 1.33) | 0.622 |  | 1.03(0.74, 1.42) | 0.849 |
| Poor |  | 1.95(0.91, 4.16) | 0.084 |  | 0.99(0.30, 3.27) | 0.991 |  | 4.07(1.56, 10.61)^**^ | 0.004 |
| **BMI(kg/m^2^)** |  |  |  |  |  |  |  |  |  |
| **<25** |  | (n=6937) |  |  | (n=6925) |  |  | (n=6934) |  |
| Good |  | Reference |  |  | Reference |  |  | Reference |  |
| Moderate |  | 1.14(0.99, 1.31) | 0.059 |  | 1.15(0.98, 1.35) | 0.092 |  | 1.09(0.86, 1.40) | 0.473 |
| Poor |  | 0.59(0.30, 1.19) | 0.144 |  | 0.51(0.22, 1.21) | 0.126 |  | 0.72(0.26, 2.04) | 0.539 |
| **≥25** |  | (n=2813) |  |  | (n=2807) |  |  | (n=2810) |  |
| Good |  | Reference |  |  | Reference |  |  | Reference |  |
| Moderate |  | 1.15(0.96, 1.38) | 0.118 |  | 1.18(0.96, 1.45) | 0.122 |  | 1.32(0.97, 1.80) | 0.079 |
| Poor |  | 1.50(0.71, 3.15) | 0.287 |  | 1.47(0.67, 3.21) | 0.337 |  | 1.43(0.41, 5.04) | 0.578 |
| **Hypertension** |  |  |  |  |  |  |  |  |  |
| **without** |  | (n=6188) |  |  | (n=6179) |  |  | (n=6183) |  |
| Good |  | Reference |  |  | Reference |  |  | Reference |  |
| Moderate |  | 1.13(0.96, 1.33) | 0.130 |  | 1.08(0.89, 1.30) | 0.440 |  | 1.32(0.98, 1.77)^*^ | 0.066 |
| Poor |  | 0.74(0.35, 1.55) | 0.423 |  | 0.51(0.18, 1.44) | 0.206 |  | 1.53(0.59, 3.99) | 0.386 |
| **with** |  | (n=3562) |  |  | (n=3553) |  |  | (n=3561) |  |
| Good |  | Reference |  |  | Reference |  |  | Reference |  |
| Moderate |  | 1.16(1.00, 1.35)^*^ | 0.049 |  | 1.24(1.04, 1.48)^*^ | 0.015 |  | 1.08(0.84, 1.38) | 0.561 |
| Poor |  | 1.00(0.51, 1.94) | 0.994 |  | 1.12(0.56, 2.23) | 0.753 |  | 0.59(0.17, 2.05) | 0.408 |
| **Diabetes** |  |  |  |  |  |  |  |  |  |
| **without** |  | (n=8685) |  |  | (n=8670) |  |  | (n=8680) |  |
| Good |  | Reference |  |  | Reference |  |  | Reference |  |
| Moderate |  | 1.12(1.00, 1.27) | 0.058 |  | 1.13(0.99, 1.30) | 0.078 |  | 1.16(0.94, 1.43) | 0.175 |
| Poor |  | 0.84(0.48, 1.44) | 0.522 |  | 0.77(0.41, 1.45) | 0.423 |  | 0.89(0.36, 2.24) | 0.812 |
| **with** |  | (n=1065) |  |  | (n=1062) |  |  | (n=1064) |  |
| Good |  | Reference |  |  | Reference |  |  | Reference |  |
| Moderate |  | 1.26(0.95, 1.66) | 0.103 |  | 1.26(0.91, 1.74) | 0.158 |  | 1.25(0.79, 1.98) | 0.339 |
| Poor |  | 0.91(0.25, 3.29) | 0.886 |  | 0.96(0.23, 3.95) | 0.950 |  | 0.83(0.15, 4.54) | 0.833 |
| **Dyslipidemia** |  |  |  |  |  |  |  |  |  |
| **without** |  | (n=6631) |  |  | (n=6617) |  |  | (n=6629) |  |
| Good |  | Reference |  |  | Reference |  |  | Reference |  |
| Moderate |  | 1.17(1.01, 1.35)^*^ | 0.031 |  | 1.17(1.00, 1.38) | 0.052 |  | 1.21(0.94, 1.56) | 0.143 |
| Poor |  | 0.87(0.48, 1.59) | 0.657 |  | 0.77(0.38, 1.53) | 0.453 |  | 1.16(0.47, 2.90) | 0.743 |
| **with** |  | (n=3119) |  |  | (n=3115) |  |  | (n=3115) |  |
| Good |  | Reference |  |  | Reference |  |  | Reference |  |
| Moderate |  | 1.10(0.93, 1.31) | 0.265 |  | 1.13(0.92, 1.38) | 0.259 |  | 1.12(0.83, 1.50) | 0.452 |
| Poor |  | 0.96(0.39, 2.35) | 0.929 |  | 1.04(0.39, 2.80) | 0.932 |  | 0.44(0.07, 2.95) | 0.398 |
| **Kidney disease**^b^ |  |  |  |  |  |  |  |  |  |
| **without** |  | (n=9223) |  |  | (n=9207) |  |  | (n=9217) |  |
| Good |  | Reference |  |  | Reference |  |  | Reference |  |
| Moderate |  | 1.13(1.01, 1.27)^*^ | 0.031 |  | 1.14(1.00, 1.30) | 0.055 |  | 1.17(0.96, 1.42) | 0.123 |
| Poor |  | 0.91(0.55, 1.51) | 0.711 |  | 0.84(0.47, 1.51) | 0.561 |  | 1.00(0.45, 2.22) | 0.991 |
| **with** |  | (n=527) |  |  | (n=525) |  |  | (n=527) |  |
| Good |  | Reference |  |  | Reference |  |  | Reference |  |
| Moderate |  | 1.38(0.93, 2.04) | 0.107 |  | 1.53(0.98, 2.38) | 0.062 |  | 1.14(0.48, 2.75) | 0.764 |
| Poor |  | 0.52(0.05, 4.89) | 0.566 |  | 0.62(0.06, 6.18) | 0.687 |  | - | - |

1. Model 4 was adjusted for age, sex, residence, education level, smoking, drinking, overweight, SBP, DBP, comorbidities and medication use.
2. Due to insufficient outcomes, the hazard ratio comparing poor balance ability to good balance ability for stroke events cannot be assessed in the population with kidney disease.

* *P*<0.05; ** *P*<0.01; *** *P*<0.001.

Abbreviations: CVD, cardiovascular disease; HR, hazard ratio; 95% CI, 95% confidence interval; BMI, body mass index; SBP, systolic blood pressure; DBP, diastolic blood pressure.

| Table S6. Model 5: Stratification analysis between baseline balance test and incident CVD, 2011-2018^a^ | | | | | | | | | |
| --- | --- | --- | --- | --- | --- | --- | --- | --- | --- |
| Stratification analysis |  | CVD | |  | Heart disease | |  | Stroke | |
|  |  | HR (95% CI) | P-value |  | HR (95% CI) | P-value |  | HR (95% CI) | P-value |
| **Total population** |  | (n=9750) |  |  | (n=9750) |  |  | (n=9750) |  |
| Good |  | Reference |  |  | Reference |  |  | Reference |  |
| Moderate |  | 1.14(1.02, 1.27)^*^ | 0.017 |  | 1.15(1.01, 1.31)^*^ | 0.030 |  | 1.15(0.95, 1.40) | 0.154 |
| Poor |  | 0.95(0.58, 1.57) | 0.840 |  | 0.89(0.50, 1.57) | 0.682 |  | 1.05(0.48, 2.32) | 0.897 |
| **Age** |  |  |  |  |  |  |  |  |  |
| **<60 years** |  | (n=5866) |  |  | (n=5858) |  |  | (n=5861) |  |
| Good |  | Reference |  |  | Reference |  |  | Reference |  |
| Moderate |  | 1.15(0.98, 1.36) | 0.090 |  | 1.16(0.96, 1.40) | 0.127 |  | 1.06(0.77, 1.46) | 0.729 |
| Poor |  | 1.58(0.64, 3.94) | 0.360 |  | 1.44(0.51, 4.06) | 0.488 |  | 2.02(0.56, 7.30) | 0.283 |
| **≥60 years** |  | (n=3884) |  |  | (n=3874) |  |  | (n=3883) |  |
| Good |  | Reference |  |  | Reference |  |  | Reference |  |
| Moderate |  | 1.13(0.98, 1.31) | 0.096 |  | 1.14(0.96, 1.36) | 0.130 |  | 1.24(0.97, 1.59) | 0.085 |
| Poor |  | 0.84(0.46, 1.54) | 0.579 |  | 0.83(0.42 1.64) | 0.591 |  | 0.82(0.29, 2.31) | 0.703 |
| **Gender** |  |  |  |  |  |  |  |  |  |
| **Male** |  | (n=4788) |  |  | (n=4780) |  |  | (n=4784) |  |
| Good |  | Reference |  |  | Reference |  |  | Reference |  |
| Moderate |  | 1.06(0.88, 1.28) | 0.545 |  | 0.95(0.75, 1.20) | 0.656 |  | 1.25(0.94, 1.67) | 0.125 |
| Poor |  | 2.62(1.18, 5.78)^*^ | 0.017 |  | 1.55(0.50, 4.80) | 0.443 |  | 5.05(1.70, 14.98)^**^ | 0.004 |
| **Female** |  | (n=4962) |  |  | (n=4952) |  |  | (n=4960) |  |
| Good |  | Reference |  |  | Reference |  |  | Reference |  |
| Moderate |  | 1.20(1.04, 1.37)^*^ | 0.010 |  | 1.27(1.09, 1.49)^**^ | 0.002 |  | 1.08(0.83, 1.41) | 0.580 |
| Poor |  | 0.76(0.42, 1.38) | 0.369 |  | 0.85(0.44, 1.63) | 0.630 |  | 0.47(0.14, 1.52) | 0.208 |
| **Residence** |  |  |  |  |  |  |  |  |  |
| **Urban** |  | (n=3402) |  |  | (n=3395) |  |  | (n=3399) |  |
| Good |  | Reference |  |  | Reference |  |  | Reference |  |
| Moderate |  | 1.04(0.86, 1.26) | 0.677 |  | 1.01(0.80, 1.27) | 0.942 |  | 1.32(0.97, 1.80) | 0.072 |
| Poor |  | 0.81(0.35, 1.87) | 0.617 |  | 0.74(0.28, 1.96) | 0.546 |  | 0.93(0.26, 3.35) | 0.912 |
| **Rural** |  | (n=6348) |  |  | (n=6337) |  |  | (n=6345) |  |
| Good |  | Reference |  |  | Reference |  |  | Reference |  |
| Moderate |  | 1.19(1.04, 1.37)^*^ | 0.010 |  | 1.23(1.05, 1.44)^**^ | 0.009 |  | 1.03(0.81, 1.33) | 0.789 |
| Poor |  | 1.04(0.56, 1.96) | 0.893 |  | 0.97(0.47, 2.01) | 0.943 |  | 1.06(0.38, 2.95) | 0.904 |
| **Smoking** |  |  |  |  |  |  |  |  |  |
| **Never** |  | (n=5802) |  |  | (n=5790) |  |  | (n=5799) |  |
| Good |  | Reference |  |  | Reference |  |  | Reference |  |
| Moderate |  | 1.22(1.07, 1.40)^**^ | 0.003 |  | 1.23(1.06, 1.44)^**^ | 0.008 |  | 1.29(1.01, 1.66)^*^ | 0.045 |
| Poor |  | 0.82(0.46 1.46) | 0.491 |  | 0.82(0.43, 1.56) | 0.553 |  | 0.71(0.25, 2.02) | 0.518 |
| **Ever/current** |  | (n=3948) |  |  | (n=3942) |  |  | (n=3945) |  |
| Good |  | Reference |  |  | Reference |  |  | Reference |  |
| Moderate |  | 1.03(0.85, 1.26) | 0.732 |  | 1.04(0.83, 1.31) | 0.715 |  | 1.00(0.72, 1.38) | 0.993 |
| Poor |  | 2.22(0.89, 5.53) | 0.086 |  | 1.75(0.52, 5.88) | 0.366 |  | 3.43(1.01, 11.63)^*^ | 0.048 |
| **Drinking** |  |  |  |  |  |  |  |  |  |
| **Never** |  | (n=5814) |  |  | (n=5802) |  |  | (n=5811) |  |
| Good |  | Reference |  |  | Reference |  |  | Reference |  |
| Moderate |  | 1.22(1.07, 1.40)^**^ | 0.003 |  | 1.21(1.03, 1.41)^*^ | 0.017 |  | 1.23(0.96, 1.59) | 0.104 |
| Poor |  | 0.77(0.41, 1.44) | 0.418 |  | 0.92(0.48, 1.74) | 0.795 |  | 0.37(0.09, 1.55) | 0.175 |
| **Ever/current** |  | (n=3936) |  |  | (n=3930) |  |  | (n=3933) |  |
| Good |  | Reference |  |  | Reference |  |  | Reference |  |
| Moderate |  | 1.02(0.84, 1.24) | 0.858 |  | 1.06(0.84, 1.33) | 0.624 |  | 1.05(0.77, 1.45) | 0.744 |
| Poor |  | 1.94(0.90, 4.16) | 0.089 |  | 0.98(0.30, 3.21) | 0.972 |  | 4.04(1.54, 10.55)^**^ | 0.004 |
| **BMI(kg/m^2^)** |  |  |  |  |  |  |  |  |  |
| **<25** |  | (n=6937) |  |  | (n=6925) |  |  | (n=6934) |  |
| Good |  | Reference |  |  | Reference |  |  | Reference |  |
| Moderate |  | 1.14(0.99, 1.31) | 0.062 |  | 1.14(0.97, 1.35) | 0.102 |  | 1.07(0.83, 1.37) | 0.611 |
| Poor |  | 0.68(0.34, 1.35) | 0.358 |  | 0.58(0.25, 1.37) | 0.215 |  | 0.87(0.31, 2.41) | 0.785 |
| **≥25** |  | (n=2813) |  |  | (n=2807) |  |  | (n=2810) |  |
| Good |  | Reference |  |  | Reference |  |  | Reference |  |
| Moderate |  | 1.15(0.96, 1.38) | 0.126 |  | 1.17(0.95, 1.45) | 0.134 |  | 1.32(0.97, 1.80) | 0.075 |
| Poor |  | 1.47(0.69, 3.13) | 0.313 |  | 1.43(0.65, 3.16) | 0.376 |  | 1.46(0.41, 5.11) | 0.558 |
| **Hypertension** |  |  |  |  |  |  |  |  |  |
| **without** |  | (n=6188) |  |  | (n=6179) |  |  | (n=6183) |  |
| Good |  | Reference |  |  | Reference |  |  | Reference |  |
| Moderate |  | 1.13(0.96, 1.33) | 0.130 |  | 1.08(0.90, 1.30) | 0.424 |  | 1.32(0.98, 1.78) | 0.069 |
| Poor |  | 0.77(0.36, 1.61) | 0.483 |  | 0.54(0.19, 1.51) | 0.238 |  | 1.60(0.61, 4.17) | 0.341 |
| **with** |  | (n=3562) |  |  | (n=3553) |  |  | (n=3561) |  |
| Good |  | Reference |  |  | Reference |  |  | Reference |  |
| Moderate |  | 1.15(0.99, 1.34) | 0.066 |  | 1.23(1.03, 1.47)^*^ | 0.023 |  | 1.04(0.81, 1.35) | 0.744 |
| Poor |  | 1.14(0.58, 2.23) | 0.697 |  | 1.27(0.63, 2.53) | 0.503 |  | 0.71(0.21, 2.43) | 0.583 |
| **Diabetes** |  |  |  |  |  |  |  |  |  |
| **without** |  | (n=8685) |  |  | (n=8670) |  |  | (n=8680) |  |
| Good |  | Reference |  |  | Reference |  |  | Reference |  |
| Moderate |  | 1.11(0.99, 1.26) | 0.081 |  | 1.12(0.97, 1.29) | 0.113 |  | 1.13(0.91, 1.40) | 0.269 |
| Poor |  | 0.88(0.51, 1.52) | 0.646 |  | 0.82(0.43, 1.53) | 0.524 |  | 0.95(0.38, 2.40) | 0.920 |
| **with** |  | (n=1065) |  |  | (n=1062) |  |  | (n=1064) |  |
| Good |  | Reference |  |  | Reference |  |  | Reference |  |
| Moderate |  | 1.26(0.95, 1.67) | 0.111 |  | 1.25(0.90, 1.74) | 0.183 |  | 1.21(0.77, 1.92) | 0.404 |
| Poor |  | 1.16(0.32, 4.21) | 0.820 |  | 1.19(0.29, 4.85) | 0.813 |  | 1.04(0.19, 5.51) | 0.967 |
| **Dyslipidemia** |  |  |  |  |  |  |  |  |  |
| **without** |  | (n=6631) |  |  | (n=6617) |  |  | (n=6629) |  |
| Good |  | Reference |  |  | Reference |  |  | Reference |  |
| Moderate |  | 1.15(1.00, 1.33)^*^ | 0.049 |  | 1.16(0.98, 1.36) | 0.082 |  | 1.17(0.90, 1.52) | 0.243 |
| Poor |  | 0.96(0.53, 1.75) | 0.903 |  | 0.84(0.42, 1.68) | 0.626 |  | 1.34(0.54, 3.32) | 0.528 |
| **with** |  | (n=3119) |  |  | (n=3115) |  |  | (n=3115) |  |
| Good |  | Reference |  |  | Reference |  |  | Reference |  |
| Moderate |  | 1.11(0.93, 1.32) | 0.230 |  | 1.13(0.92, 1.39) | 0.245 |  | 1.13(0.84, 1.52) | 0.410 |
| Poor |  | 0.99(0.40, 2.42) | 0.979 |  | 1.07(0.40, 2.85) | 0.899 |  | 0.47(0.07, 3.10) | 0.431 |
| **Kidney disease**^b^ |  |  |  |  |  |  |  |  |  |
| **without** |  | (n=9223) |  |  | (n=9207) |  |  | (n=9217) |  |
| Good |  | Reference |  |  | Reference |  |  | Reference |  |
| Moderate |  | 1.13(1.00, 1.26)^*^ | 0.042 |  | 1.13(0.99, 1.29) | 0.080 |  | 1.15(0.94, 1.40) | 0.187 |
| Poor |  | 1.00(0.61, 1.66) | 0.990 |  | 0.92(0.52, 1.65) | 0.788 |  | 1.15(0.52, 2.54) | 0.727 |
| **with** |  | (n=527) |  |  | (n=525) |  |  | (n=527) |  |
| Good |  | Reference |  |  | Reference |  |  | Reference |  |
| Moderate |  | 1.39(0.94, 2.06) | 0.101 |  | 1.58(1.00, 2.49)^*^ | 0.049 |  | 1.23(0.49, 3.12) | 0.659 |
| Poor |  | 0.46(0.04, 4.92) | 0.522 |  | 0.55(0.05, 6.02) | 0.626 |  | - | - |

1. Model 5 was adjusted for age, sex, residence, education level, smoking, drinking, overweight, SBP, DBP, comorbidities, medication use and metabolic biomarkers.
2. Due to insufficient outcomes, the hazard ratio comparing poor balance ability to good balance ability for stroke events cannot be assessed in the population with kidney disease.

* *P*<0.05; ** *P*<0.01; *** *P*<0.001.

Abbreviations: CVD, cardiovascular disease; HR, hazard ratio; 95% CI, 95% confidence interval; BMI, body mass index; SBP, systolic blood pressure; DBP, diastolic blood pressure.
